# Home-based cognitive training in pediatric patients with acquired brain injury: preliminary results on efficacy of a randomized clinical trial

**DOI:** 10.1038/s41598-020-57952-5

**Published:** 2020-01-29

**Authors:** Claudia Corti, Cosimo Urgesi, Geraldina Poggi, Sandra Strazzer, Renato Borgatti, Alessandra Bardoni

**Affiliations:** 1 Scientific Institute, IRCCS E. Medea, Bosisio Parini, Lecco, Italy; 2 Scientific Institute, IRCCS E. Medea, San Vito al Tagliamento, Pordenone, Italy; 3 University of Udine, Laboratory of Cognitive Neuroscience, Department of Languages and Literatures, Communication, Education and Society, Udine, Italy

**Keywords:** Neuroscience, Brain injuries

## Abstract

Cognitive rehabilitation may compensate for cognitive deficits of children with acquired brain injury (ABI), capitalizing on the use-dependent plasticity of a developing brain. Remote computerized cognitive training (CCT) may be delivered to patients in ecological settings, ensuring rehabilitation continuity. This work evaluated cognitive and psychological adjustment outcomes of an 8-week multi-domain, home-based CCT (Lumosity Cognitive Training) in a sample of patients with ABI aged 11–16 years. Two groups of patients were engaged in five CCT sessions per week for eight weeks (40 sessions). According to a stepped-wedge research design, one group (Training-first Group) started the CCT immediately, whereas the other group (Waiting-first Group) started the CCT after a comparable time of waiting list. Changes after the training and after the waiting period were compared in the two groups. Both groups improved in visual-spatial working memory more after the training than after the waiting-list period. The Training-first group improved also in arithmetic calculation speed. Findings indicate that a multi-domain CCT can produce benefits in visual-spatial working memory, probably because, in accordance with previous research, computer games heavily tax visuo-spatial abilities. This suggests that the prolonged stimulation of the same cognitive ability may generate the greatest benefits in children with ABI.

## Introduction

Pediatric acquired brain injuries (ABI) are among the main causes of lifelong disabilities in school age children and are often accompanied by cognitive, behavioral and affective problems^1–4^. From a cognitive point of view, impairments in global intelligence or core cognitive domains, such as attention, memory, executive functions, processing speed and visual abilities, have been reported^2,3^. These impairments may be associated with negative academic outcomes and short- and long-term functional problems at home and in general life in community^5–7^. Even those children having an intellectual quotient within the normal range often experience difficulties at school after the injury^8,9^. Tailored rehabilitation on cognitive functions has been recognized to be a key component of medical care following an ABI, as it may lead to improved functioning in everyday life^10–16^. Indeed, early stimulation of cognitive functions may promote use-dependent brain plasticity^17,18^ and enhance the potential for the inherent brain plasticity after damage^19^. However, after hospitalization, a high number of children with ABI do not receive any help or support, experiencing a problematic return to ecological settings^20,21^.

Recently, technological devices have been introduced in rehabilitation to provide treatments deliverable in ecological settings with the aim to overcome the limitations of a traditional rehabilitative approach, such as elevated costs, accessibility problems and heterogeneity in treatment practice, and to reach larger numbers of patients^22–24^. The majority of these programs have been developed on computerized platforms, allowing the delivery of more engaging exercises compared to traditional paper-and-pencil tasks^22–24^. Thus far, positive results on the feasibility and accessibility of remote computerized cognitive training (CCT) for children with ABI have been documented^24,25^. Preliminary results on the efficacy of CCT in these children^14–16,26^ or in children with neurodevelopmental disorders^27,28^ have also been provided. A recent meta-analysis indicated that remote CCT programs based on the repetition of cognitive exercises (drill-based exercises) have an effect on the visual-spatial abilities of children with ABI, but no effects were documented for other cognitive abilities^26^. Nevertheless, evidence of the effects on far transfer measures of CCT programs for children with ABI has been questioned^29,30^. Overall, contrasting results on CCT efficacy have been reported in the literature and no clear indication of which training format is optimal (i.e., training performed under the guidance of a healthcare practitioner or independently; training intensity and duration; one- or multi-domain stimulation etc.) to enhance cognitive performance has been provided. In particular, it is unclear whether the active contribution of a therapist on cognitive performance (e.g., providing a feedback, suggesting strategies, and correcting errors) is required for obtaining positive CCT effects on neurocognitive outcomes. Gathering information on the abilities which could be remotely improved by a CCT without the intervention of a clinician might potentiate and extend the usefulness of CCT programs delivered in ecological settings. Furthermore, no certain indication on the ideal type of cognitive stimulation provided by the CCT to enhance cognitive abilities, namely single-domain on multi-domain stimulation, exists. However, in view of the interdependence of different cognitive abilities^31,32^ and based on previous studies on CCT conducted on healthy individuals^33–37^, the simultaneous stimulation of different cognitive domains by the same training is expected to generate the greatest impact on cognitive outcomes and produce improvement across untrained cognitive domains. This effect was also found in adults with chronic mild-to-severe traumatic brain injury^38^ but this still needs to be verified in pediatric individuals with ABI.

This study aimed at testing, in a group of pediatric patients with non-progressive ABI, the effects of a remotely-delivered, multi-domain CCT, namely Lumosity Cognitive Training (Lumos Labs, Inc.), on the neurocognitive functioning and psychological adjustment. Lumosity Cognitive Training is a commercially available CCT designed to boost cognitive abilities in the general population^39^, without specific clinical aims. However, a previous study of the CCT conducted by our research group has documented its feasibility and acceptability in children with acquired or congenital brain injury, irrespective of participants’ intellectual abilities^24^. Indeed, out of a sample of 32 patients (average full scale intellectual quotient (FSIQ) = 89), 93.55% of participants completed at least 90% of the training program (which was composed of 40 sessions) in the given timeframe of 8 weeks. Most patients positively evaluated the usefulness and pleasantness of the CCT and we had no drop-outs at follow-up assessments among patients who completed the CCT, thus indicating a limited risk of attrition bias for subsequent analyses on efficacy. Moreover, a previous study on Lumosity Cognitive Training addressing executive function skills in children with brain tumor found near- and far-transfer effects from this training on different neurocognitive abilities^40^, pointing to a promising opportunity to use the CCT in the rehabilitation of children with ABI.

The current study is part of a randomized clinical trial on the feasibility and efficacy of such a CCT in children and adolescents with either congenital or acquired brain injury (see Supplementary Information 1). However, in addition to estimate the overall effect of the CCT in the wide population of patients with brain injury, the trial also aimed to test the effects of the CCT in different subgroups of patients based on a specific diagnosis, with the goal of studying how specific brain injured populations react to training administration and benefit from it. Therefore, for this study, children with non-progressive ABI only (traumatic brain injury (TBI), ischemic or hemorrhagic lesion, anoxia and central nervous system infections) were selected. This choice was motivated by the fact that previous literature reported differences in functional outcomes and treatment effects according to the etiology of the brain damage (i.e., congenital or acquired)^41^ and to its development (i.e., progressive, such as brain tumor, or non-progressive, such as TBI, vascular or infectious brain lesions)^42^, more than to the specific diagnosis (i.e., anoxia vs. TBI^43^; stroke vs. TBI^44^). This study reports the subgroup analysis of the effects of the CCT on non-progressive ABI, whose recruitment was completed by the 31^st^ December 2017. The remaining part of the trial was dedicated at concluding recruitment of children with progressive ABI and congenital brain injury (recruitment end date: 27/11/2019; main trial overall end date: 27/09/2020), whose subgroup analysis results, along with the overall results on the wider population of pediatric patients with brain injury, will be published in subsequent reports. Since no further patients with non-progressive ABI will be added to this subgroup analysis and recruitment of patients with a different diagnosis of brain injury has stopped, this preliminary report does not suffer from the risks associated with publication of interim analyses, namely differences in effect estimates as compared to the final report^45^ or bias in the ongoing recruitment of patients^46^. It rather allows for prompt publication of trial results, in keeping with the trial dissemination plan^47^, as soon as they are available^48^.

**Figure 1:**
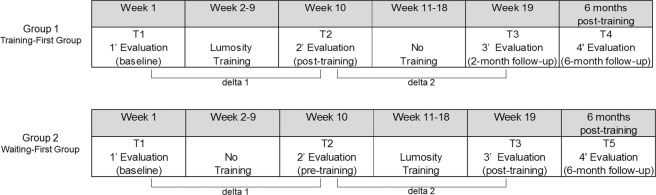
Study design.

The study used a randomized, stepped-wedge design (Fig. 1): participants of one group (Training-first Group) started the 8-week CCT after a baseline assessment, received the post-training evaluation and then waited a comparable amount of time before the third assessment; participants of the other group (Waiting-first Group) were allocated to an 8-week waiting-list period after the baseline assessment, received the second evaluation, then started the training and finally received the third assessment, which corresponded to post-training evaluation. The adoption of such a design allowed for isolating the training-specific effects over any learning effects that could be observed across assessment sessions. For the CCT used in this study, five games stimulating five different core cognitive abilities (memory, attention, cognitive flexibility, speed of processing and math problem-solving) were selected from the pool of exercises available from Lumosity Cognitive Training. The choice to select games stimulating different neurocognitive abilities was made with the goal to target the different cognitive impairments of children and adolescents with ABI and to foster transferability of the potential cognitive improvements to everyday-life functioning. Accordingly, training efficacy was evaluated both on neurocognitive domains addressed by the program utilizing tasks different than those practiced in the CCT (near-transfer effects on tasks different from the training) and on psychological adjustment (far-transfer effects).

It was hypothesized that visual-spatial working memory could be improved after the training (primary outcome) for the following reasons: i) the CCT provided exercises addressing different cognitive skills but all strongly relying on visual-spatial abilities; ii) previous research found benefits in visual-related abilities from videogames^49–51^ in the healthy population and the CCT exercises had a game-like format; iii) the CCT proposed drill-based exercises and previous research found that CCT programs mostly based on drill-based exercises produce gains in visual-spatial abilities of children with ABI. In contrast, there is more limited evidence on the benefits of CCT programs based on drill-based exercises on cognitive abilities different from visual-spatial skills in either the general population or in children and adolescents with ABI. Therefore, as an exploratory hypothesis, we evaluated training effects on the other trained cognitive domains (secondary outcomes). Finally, based on previous research, the improvement in working memory and other cognitive abilities was expected to also impact on behavioral aspects and psychological health^52–54^, with transfer of benefits to everyday-life functioning (far-transfer outcomes).

## Results

### Recruited patients

**Figure 2:**
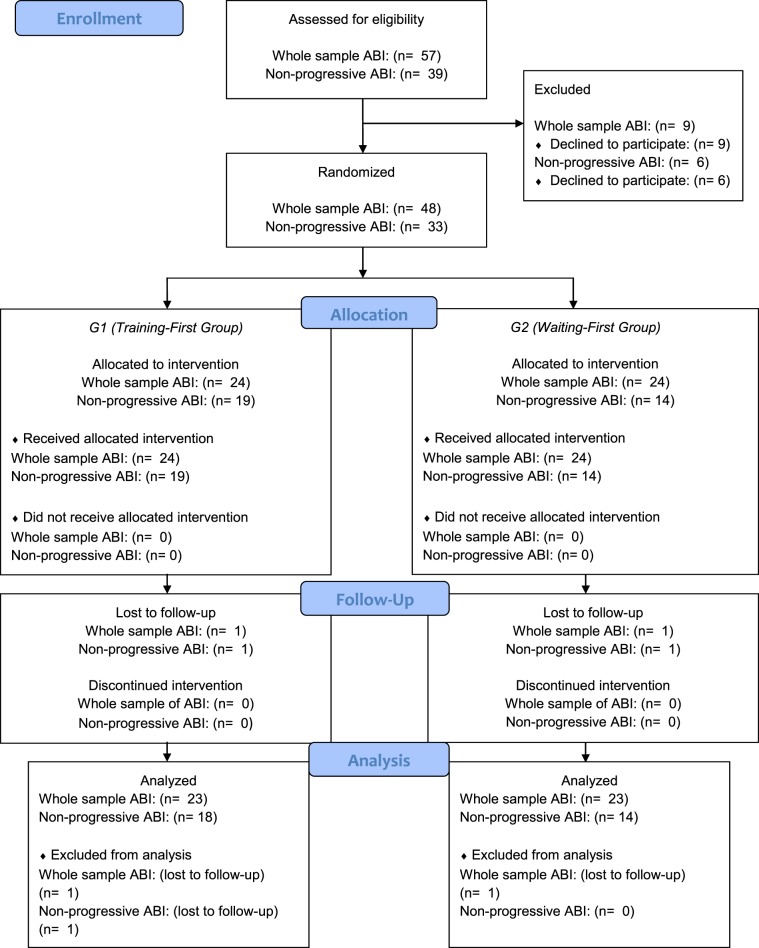
Study flowchart depicting the number of patients with acquired brain injury (ABI) collected for every research step. Note. As in this study only patients with non-progressive ABI were selected from the whole sample of patients with ABI, the flowchart shows the number of patients with ABI of both the main study (“Whole sample ABI”, including both progressive and non-progressive ABI) and of the present study (“Non-progressive ABI”). The category “Non-progressive ABI” includes injuries associated with stroke, traumatic brain injury, anoxia, meningitis, encephalitis, post-surgical meningioma and acoustic neuroma, whilst it excludes brain damage due to tumors presenting the possibility of illness degeneration and/or of progressive neuroanatomical damage associated with adjuvant therapies.

At 31 December 2017, 33 patients included in the main study met the inclusion criteria for this study. Among them, 19 subjects were allocated to the Training-first Group (G1) and 14 to the Waiting-first Group (G2). One patient in the Training-first Group revoked participation from the study and was lost at post-training evaluations, so that the final number of participants for whom statistical analyses were conducted was of 32 patients. Out of them, 18 participants were allocated to the Training-first Group and 14 to the Waiting-first Group. The flowchart of the current study is presented below (Fig. 2). As depicted in the flowchart, the number of children assigned to the Training-first Group and Waiting-first Group was different, as for the main study participants were stratified based on etiology of the brain injury (acquired or congenital), but not on the classification of progressive/non-progressive ABI.

Nevertheless, no differences in demographic, clinical and intellectual variables (Table 1) or in cognitive functioning with respect to the domains addressed by the training and psychological functioning (Table 2) were found at baseline assessment between the Training first Group (G1) and the Waiting-first Group (G2). With respect to demographic characteristics, no differences were found for gender (*χ*^2^(31) = 0.55; p = 0.46), age (t(30) = 0.52; P = 0.61) and socio-economic status (SES), as assessed through Holligshead’s classification^55^, (t(30) = −1.05; P = 0.30). In relation to intellectual and cognitive functioning, no differences were found in the following measures: FSIQ^56^ (t(30) = 0.41; P = 0.69), visual-spatial working memory^57^ (t(30) = −0.19; P = 0.85), cognitive flexibility^58^ (t(30) = −0.95; P = 0.35), arithmetic calculation-accuracy^59–61^ (t(30) = 0.50; P = 0.62), arithmetic calculation-speed^59–61^ (t(30) = 0.35; P = 0.73) and problem-solving^59–61^ (t(30) = 0.17; P = 0.87). Similarly, no differences were observed in relation to psychological measures, as assessed through the Child Behavior Checklist (CBCL)^62^: CBCL internalizing (t(30) = 0.54; P = 0.60), CBCL externalizing (t(30) = −0.36; P = 0.72) and CBCL Total Score (t(30) = 0.23; P = 0.82). With respect to clinical characteristics, the most common diagnosis was brain trauma (62.5%), followed by ischemic and hemorrhagic lesion (28.1%), anoxia (6.3%) and encephalitis (3.1%). No differences in specific diagnosis distribution (*χ*^2^(3) = 2.86, P = 0.41) and injury severity level (t(30) = −0.07, P = 0.94) as assessed through Glasgow Coma Scale (GCS)^63^ were found. This confirmed randomization and ruled out that any differences between the two groups were due to their baseline performance at study entry.

**Table 1:** Demographic, clinical and intellectual characteristics of participants (Group 1 and Group 2) at baseline.

	Training-first Group (G1) (n = 18)		Waiting-first Group (G2) (n = 14)	
	M(SD)/n(%)		M(SD)/n(%)	
**Demographic variables**				
Gender (male)	12	(66.70%)	11	(78.60%)
Age (years)	13.83	(1.65)	13.50	(1.99)
SES	5.22	(1.73)	4.57	(1.72)
**Clinical variables**				
Diagnosis				
*TBI*	11	(61.10%)	9	(64.30%)
*Stroke*	5	(27.80%)	4	(28.60%)
*Anoxia*	2	(11.10%)	0	(0.00%)
*Encephalitis*	0	(0.00%)	1	(71.00%)
Injury severity level (GCS)				
*Severe*	14	(77.80%)	11	(78.60%)
*Moderate*	2	(11.10%)	2	(14.30%)
*Mild*	2	(11.10%)	1	(71.00%)
**Intellectual functioning**				
FSIQ	88.39	(18.44)	84.93	(29.53)

Note. FSIQ = Full Scale Intellectual Quotient; GCS = Glasgow Coma Scale; SES = socio-economic status; TBI = traumatic brain injury.

**Table 2:** Means and standard deviations of standardized test scores for each cognitive and psychological outcome measure of Group 1 and Group 2 at T1, T2 and T3.

	Training-first Group (G1) (n = 18)						Waiting-first Group (G2) (n = 14)					
	M(SD)						M(SD)					
	T1		T2		T3		T1		T2		T3	
**Cognitive functioning**												
Visual-spatial working memory^z^	−0.95	(1.04)	−0.10	(1.21)	−0.44	(0.97)	−0.88	(1.18)	−1.07	(1.64)	−0.73	(1.43)
Cognitive Flexibility^z^	0.53	(1.43)	0.65	(1.24)	0.57	(1.17)	0.03	(1.57)	0.02	(1.55)	0.51	(1.47)
Arithmetic calculation – accuracy^z^	−0.82	(1.64)	−0.27	(1.70)	−0.64	(1.80)	−0.85	(1.70)	−0.71	(1.75)	−0.56	(1.22)
Arithmetic calculation – speed^z^	−1.47	(1.51)	−0.90	(1.65)	−1.06	(1.83)	−2.04	(2.37)	−1.71	(2.40)	−1.19	(2.43)
Problem-solving^z^	−0.97	(1.46)	−0.74	(1.62)	−0.71	(1.49)	−1.15	(1.56)	−1.15	(1.83)	−1.09	(2.02)
**Psychological Adjustment**												
CBCL Internalizing^T^	59.00	(7.24)	54.50	(9.87)	54.56	(10.19)	57.57	(7.78)	56.79	(7.92)	57.14	(8.90)
CBCL Externalizing^T^	53.56	(9.24)	50.94	(7.67)	52.06	(6.57)	54.79	(9.81)	53.29	(9.40)	55.50	(6.78)
CBCL Total Score^T^	58.22	(7.87)	54.44	(8.71)	54.83	(7.72)	57.57	(8.25)	56.86	(7.09)	57.21	(6.65)

Note. ^z^indicates measures expressed as z-scores (M = 0, SD = 1); ^T^indicates measures expressed as T-scores (M = 50, SD = 10); CBCL = Child Behavior Checklist.

### Completed Sessions and practice-related effects

The average number of performed sessions was 39.34 (1.31), out of a defined total number of 40 sessions. No significant difference (t(30) = −1.62, P = 0.12) was found in average number of sessions between the Training-first Group (M = 39.67, SD = 0.97) and the Waiting-first Group (M = 38.93, SD = 1.59). The minimum percentage of sessions completed was 90% (36 sessions). No significant difference (t(30) = −1.00, P = 0.32) in practice-related effects (LPI-change) between the Training-first Group (M = 239.50, SD = 201.02) and the Waiting-first Group (M = 161.86, SD = 236.93) was found. No participants reported discomfort during the training and no unintended effects were detected.

### Primary cognitive outcome

**Figure 3:**
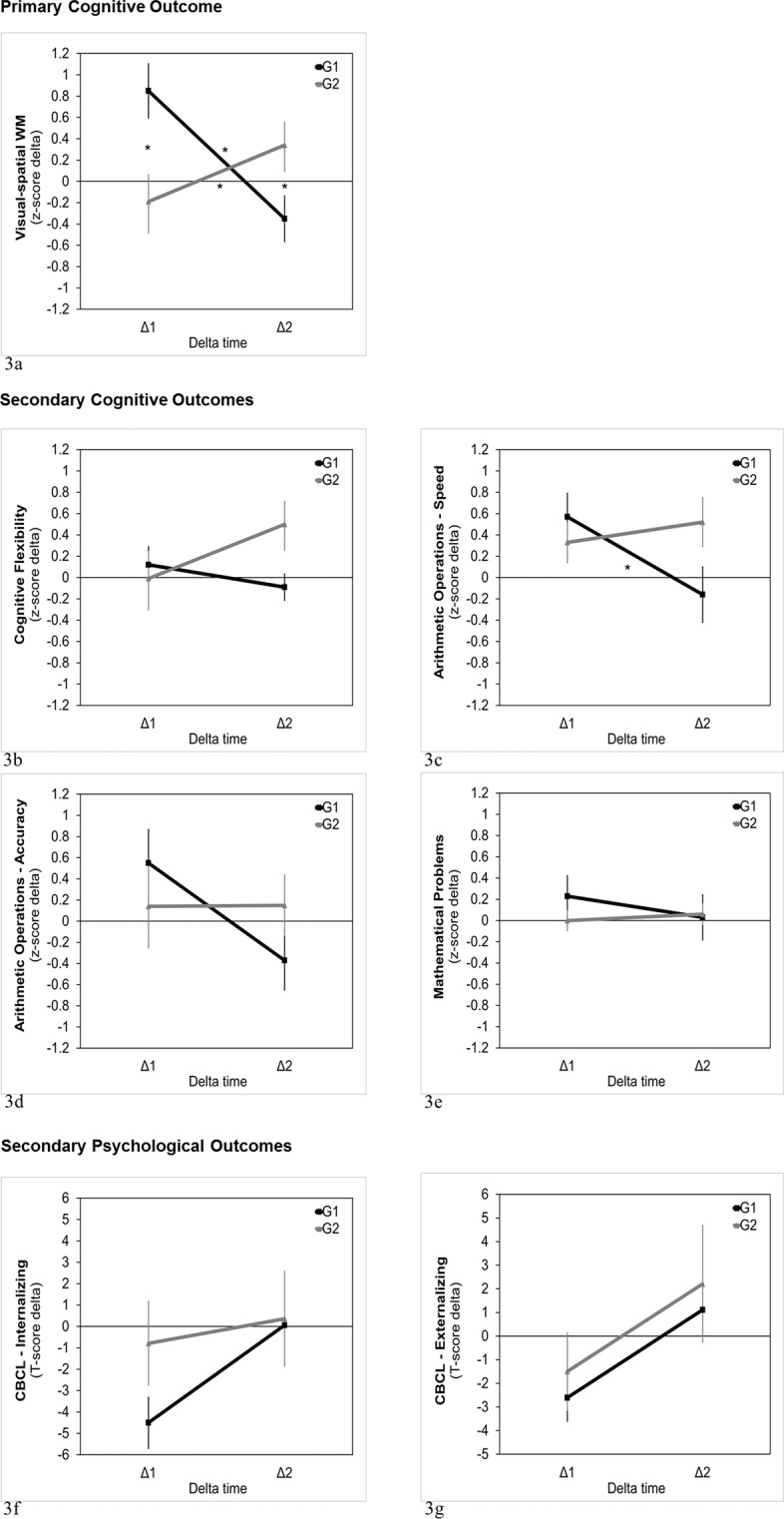
Delta change values (delta 1 and delta 2) for Group 1 (Training-first Group) and Group 2 (Waiting-firts Group) in any cognitive and psychological measures. Note. Delta 1 represents the difference in performance between T2 and T1; delta 2 represents the difference in performance between T3 and T2.

To test the near-transfer effects on cognitive measures and the far-transfer effects on psychological adjustment, we calculated the within-subject difference in performance between the second and the first evaluation (delta 1: T2-T1) and between the third and the second evaluation (delta 2: T3-T2) (Fig. 3). With respect to the primary cognitive outcome of this study, that is visual-spatial working-memory, the mixed 2-way ANOVA on this measure, with delta time (delta 1/delta 2) as within-subject variable and Group (Training-first Group/Waiting-first Group) as between-subject variable, revealed non-significant main effects of delta time (F(1, 30) = 1.10, P = 0.30, ${\eta }_{p}^{1}=0.04$) and group (F(1, 30) = 0.94, P = 0.34, ${\eta }_{p}^{2}=0.03$). Conversely, a significant interaction between delta time and group was found (F(1, 30) = 7.40, P = 0.01, ${\eta }_{p}^{2}=0.20$), with moderate to large effect-size. When controlling for the possible influence of individual intellectual ability and practice-related improvements on the trained tasks, the interaction delta time x group remained significant even when FSIQ and Lumosity Performance Index (LPI-change) were inserted as covariates (F(1, 28) = 7.94, P < 0.01, ${\eta }_{p}^{2}=0.22$). No interaction effects between FSIQ and delta time (F(1, 28) = 2.89, P = 0.10, ${\eta }_{p}^{2}=0.09$) and between LPI-change and delta time (F(1, 28) = 1.24, P = 0.28, ${\eta }_{p}^{2}=0.04$) were found.

Duncan post-hoc analyses on visual-spatial working memory revealed that, in the Training-first Group (G1), delta 1 (M = 0.85, sem = 0.26) was significantly larger than delta 2 (M = −0.35, sem = 0.22; P < 0.01), with a very large effect (Cohen’s d = 1.21); in contrast, in the Waiting-first Group (G2), the difference between delta 1 (M = −0.19, sem = 0.30) and delta 2 (M = 0.34, sem = 0.25) was marginally significant (P = 0.05) and in the expected direction of a medium-sized improvement after training (Cohen’s d = 0.53). This indicates that in both groups performance improved more after the training (delta 1 in the Training-first Group and delta 2 in the Waiting-first Group) than after a non-training condition (delta 1 in the Waiting-first Group and delta 2 in the Training-first Group). Moreover, the difference between the two groups in delta 1 and delta 2 resulted to be significant and in the expected direction, with delta 1 being larger in the Training-first Group (G1) than in the Waiting-first Group (G2) (P < 0.01; Cohen’s d = 0.97), and delta 2 being larger in the Waiting-first Group (G2) than in the Training-first Group (G1) (P = 0.04; Cohen’s d = 0.76).

### Secondary cognitive outcomes

Non-significant main effects or interaction were found in ANOVA on the following cognitive secondary outcome measures: cognitive flexibility (delta time (F(1, 30) = 0.50, P = 0.48, ${\eta }_{p}^{2}=0.02$); group (F(1, 30) = 1.61, P = 0.22, ${\eta }_{p}^{2}=0.05$); interaction (F(1, 30) = 2.74, P = 0.11, ${\eta }_{p}^{2}=0.08$)); arithmetic calculation accuracy (delta time (F(1, 30) = 0.50, P = 0.48, ${\eta }_{p}^{2}=0.05$); group (F(1, 30) = 2.12, P = 0.22, ${\eta }_{p}^{2}=0.05$); interaction (F(1, 30) = 2.74, P = 0.11, ${\eta }_{p}^{2}=0.08$)); mathematical problem-solving (delta time (F(1, 30) = 0.17, P = 0.68, ${\eta }_{p}^{2}=0.00$); group (F(1, 30) = 0.32, P = 0.57, ${\eta }_{p}^{2}=0.01$); interaction (F(1, 30) = 0.57, P = 0.46, ${\eta }_{p}^{2}=0.02$)). In contrast, for arithmetic calculation speed, the main effects of delta time (F(1, 30) = 1.47, P = 0.23, ${\eta }_{p}^{2}=0.05$) and group (F(1, 30) = 0.87, P = 0.36, ${\eta }_{p}^{2}=0.03$) were not significant, but their interaction resulted to be significant (F(1, 30) = 4.36, P < 0.05, ${\eta }_{p}^{2}=0.13$), with moderate to large effect size (Fig. 3).

Duncan post-hoc tests on arithmetic calculation speed revealed that, in the Training-first Group, delta 1 was significantly larger (M = 0.57, sem = 0.20) than delta 2 (M = −0.16, sem = 0.23; P < 0.01; Cohen’s d = 0.82), having the expected direction of an improvement after the training. Conversely, in the Waiting-first Group, the small difference between delta 1 (M = 0.33, sem = 0.22) and delta 2 (M = 0.52, sem = 0.26) was not significant (p = 0.57; Cohen’s d = 0.22), even if the latter (change after training) was positive and tended to be greater than the former (change after non-training). Indeed, the medium-sized difference between the delta 2 values of the two groups showed a trend towards statistical significance (P = 0.08; Cohen’s d = 0.72), being larger in the Waiting-first group than in the Training-first Group, as expected. No other comparisons resulted to be significant. However, the interaction delta time x group did not reach significance after controlling for the influence of FSIQ and LPI-change (F(1, 30) = 3.66, P = 0.07, ${\eta }_{p}^{2}=0.12$), suggesting that differences in general cognitive abilities and practice-related effects might mediate the differences between the two groups in arithmetic calculation speed.

### Secondary adjustment outcomes

With respect to psychological adjustment, the repeated-measure ANOVAs on CBCL scores (CBCL internalizing, CBCL externalizing and CBCL Total Score) showed non-significant main effects of delta time (F(1, 30) < 4, P > 0.06; ${\eta }_{p}^{2} < 0.12$) and group (F(1, 30) < 2.23, P = 0.15; ${\eta }_{p}^{2} < 0.07$) and non-significant interaction between delta time and group (F(1, 30) < 1.11, P > 0.30, ${\eta }_{p}^{2}=0.04$) (Fig. 3).

### Follow-up analyses

In the whole group, the difference between the change after treatment (collapsing T3 – T2, i.e. delta 2, for the Waiting-first Group and T2 – T1, i.e. delta 1, for the Training-first Group) and the spontaneous change (collapsing T3 – T2, i.e. delta 2, for the Waiting-first Group and T2 – T1, i.e. delta 1, for the Training-first Group) resulted to be significant for both visual-spatial working memory (after treatment: M = 0.63; SD = 1.10; spontaneous change: M = −0.28; SD = 0.97; t(31) = 2.87, P < 0.01, Cohen’s d = 0.89) and arithmetic calculation speed (after treatment: M = 0.50; SD = 0.87; spontaneous change: M = −0.08; SD = 0.75; t(31) = 2.99, P < 0.01, Cohen’s d = 0.72), with moderate to large effects for both variables.

For the Training-first Group, the comparison between T3 (2-month follow-up after the training) and T1 (pre-training), aimed at assessing CCT long-term effects, showed a significant long-term effect of the training on visual-spatial working memory (t(17) = 2.01, P = 0.03), but only a trend towards the expected direction for the long-term effect on arithmetic calculation speed (t(17) = 1.46, P = 0.08).

In a post-hoc power analysis, based on the significant interaction effect time x group on visual-spatial working memory found at general linear model (η_p_^2^ = 0.20), we estimated that, with a sample size of 32 subjects and an effect size of *f(V)* = 0.05, assuming a correlation of 0.50 between repeated measures and an alfa level set at P < 0.05, a power of 0.99 could be obtained. A sensitivity analysis showed that, with our sample size of 32 participants, an effect size of at least Cohen’s *d* = 0.50 (medium size) was required to observe a significant one-tailed difference between the improvement after training and the spontaneous change at an alfa level of P < 0.05 with 0.99 power.

## Discussion

This study tested the efficacy of an 8-week home-based CCT in children with non-progressive ABI using a randomized, stepped-wedge research design. Findings of this study represent the preliminary results of a still ongoing clinical trial on the feasibility and efficacy of Lumosity Cognitive Training in a sample of pediatric patients with congenital or acquired brain injury. Based on extant literature, we hypothesized near-transfer effects of the CCT on visual-spatial working-memory, as primary outcome, and far-transfer effects on psychological adjustment as secondary outcomes. Moreover, we explored near-transfer effects on the other trained cognitive abilities.

The primary hypothesis of CCT efficacy on visual-spatial working-memory was confirmed by our data. Indeed, performance in visual-spatial working-memory improved more after the training than after the waiting-list period (Waiting-first Group) or the 2-month follow-up (Training-first Group). Furthermore, the Training-first Group showed a higher level improvement at the second evaluation (post-training) than the Waiting-first Group (pre-training). This trend, however, was inverted at the third evaluation, after the Waiting-first Group was provided with the CCT: at this time point, the Waiting-first Group showed bigger improvement than the Training-first Group, which had rested for the same amount of time. The beneficial effects of the CCT on visual-spatial working memory were maintained at the 2-month follow-up, since the performance of the Training-first Group patients after the 2-month rest was still higher than their baseline evaluation before the training, thus indicating long-term benefits from the CCT. Training activities and outcome measures used to assess visual-spatial skills did not present similarities, thus indicating that the CCT improved performance in non-trained tasks (near-transfer effects on tasks different from the training).

Nevertheless, the improvement in visual-spatial working-memory could be due to the consistent stimulation of visual-spatial processes across all CCT exercises. Indeed, the five proposed games, albeit focusing on different cognitive domains, relied on visual-spatial competence as they required participants to: i) detect the orientation of a stimulus in space (Disillusion, Lost in Migration), ii) match together (Disillusion) or recognize (Tidal Treasure, Speed Match) visually presented figures that could differ in shape and color, iii) solve arithmetic operations contained in drops that moved vertically on the computer screen and were distributed in space (Raindrops), and iv) maintain in working memory the shapes and colors of visual stimuli (Tidal Treasure, Speed Match). This hypothesis is in line with the notion that computer games heavily tax visual-spatial working-memory^49–51^ and that the benefits of games like *Tetris* on mental health are likely proxied by occupying visual-spatial working memory^64–67^. In keeping with it, previous research showed that visual-spatial abilities are enhanced by video- and computer-game playing, even after a few months of training^50^.

Notably, the CCT used in this study (Lumosity Cognitive Training) was not developed with a specific rehabilitative purpose and did not require the monitoring by a therapist on cognitive performance. This suggests that the intrinsic features of the program, such as the fact that the exercises were designed by professionals with expertise in neuropsychology, their game-like format and the ability of the CCT to adapt to user’s performance, could be sufficient to provide benefits on the visual abilities of patients.

The CCT effects on visual-spatial working memory were not associated with individual FSIQ, thus suggesting that CCT programs may be effective in ABI patients with different level of cognitive impairment. Nor were the effects associated with practice-related improvement at gaming performance (LPI-change), despite the large individual variability in this parameter. However, since the LPI-change was calculated as the mean change observed across all CCT game domains, it may not have measured the specific improvement in visual-spatial skills. Thus the finding that cognitive improvement was independent from practice-related improvement on the CCT should be considered with caution.

Our exploratory hypothesis about the efficacy of the tested CCT on neuropsychological domains different from visual-spatial skills was not supported by results. Indeed, no CCT effects were observed for cognitive flexibility and the accuracy of arithmetic calculation and only small and short-lasting effects were obtained on the arithmetic calculation speed. In this latter measure, the Training-first Group showed a speed up of arithmetic calculation immediately after the training, but this improvement was not maintained at the 2-month follow-up, suggesting only short-term training effects. Moreover, this gain was not detected for the Waiting-first Group, suggesting that the improvement observed in the Training-first Group at the second evaluation may reflect the accumulative effects of training and learning. Since the very same operations were administered at all evaluations, it is possible that the learning effects were stronger when participants repeated the operations for the second time and had reached the ceiling of their performance capability for further repetitions. Thus, although the smaller sample size of the Waiting-first Group compared to the one of the Training-first Group prevents us from ruling out power issues or sampling biases, we suspect that the benefits of the CCT in the arithmetic domain, “if any”, were limited and not generalizable to all participants. To sum up, in contrast to previous findings on the efficacy of multi-domain cognitive stimulation on global cognitive performance^33–37^, our data questions the capability of a CCT to boost cognitive domains not intensively addressed by the training program. They rather suggest that the best cognitive benefits in pediatric ABI are achieved by the intensive stimulation of the same cognitive function. Indeed, in this study, while visual-spatial skills were stimulated by all training games, the other cognitive abilities were not so hardly stimulated, which could explain the failure of the CCT in improving them. While studies that reported wide cognitive gains from a multi-domain CCT had enrolled healthy individuals^33–37^, our study involved a clinical population of patients with ABI. Thus, we may hypothesize that in case of brain damage the occurrence of cognitive benefits in a specific domain may emerge only after an intensive and prolonged stimulation of domain-related abilities. This could be due to diffuse axonal injury, which is reported to be a relevant clinical feature of pediatric ABI^68^ and may contribute to reduced neural reorganization of a damaged brain compared to what occurs in a non-damaged brain, thus limiting transfer effects between cognitive domains.

Also regarding psychosocial adjustment, no significant improvement was found on both internalizing and externalizing symptoms of CBCL. This may lead to exclude possible expectation effects, as the parents of children of both the Training-first Group and Waiting-first Group provided comparable evaluation of their children at all assessment points of the stepped-wedge design. Such a finding seems to indicate that commitment into a CCT is not sufficient to lead parents to report a reduction of children’s behavioral problems, excluding the occurrence of placebo-related effects. At the same time, however, the absence of any changes in psychological adjustment seems to suggest that the CCT had no far-transfer effects in every-day life behavior. This might reflect the fact that the CCT only exercised cognitive performance and did not directly address problem solving in everyday-life situations. Similar results and interpretation were reported in a previous study^69^ involving a group of children with ABI that were offered a CCT stimulating a wide range of cognitive abilities. Cicerone and colleagues^70^ suggested that more complex metacognitive abilities (e.g., cognitive self-monitoring, emotional self-appraisal, and imagined use of strategies in real situations) should be stimulated in pediatric ABI to generate CCT effects on everyday functioning. This would explain why numerous studies found no generalization effects of CCT programs, questioning their usefulness with respect to adjustment in ecological settings^71–73^.

In conclusion, with respect to clinical implications and generalizability of findings, this study seems to support the efficacy of a remote multi-domain CCT strongly relying on visual-spatial processes in improving visual-spatial working memory of children with non-progressive ABI. The delivery of home-based interventions to enhance cognitive functioning should be considered as an important opportunity for the rehabilitation of children with non-progressive ABI. Indeed, CCT programs performed in ecological settings may limit the time and economic demands on families associated with reaching the rehabilitation centers and ensure treatment delivery to those patients living far from the hospitals^74,75^. Findings support the hypothesis that the prolonged stimulation of the same cognitive domain generates the best outcomes in these patients, calling into question the efficacy of a multi-domain stimulation in simultaneously improving different cognitive abilities. Therefore, as CCT programs are usually time-constrained, longer periods of practice may be required to stimulate at the same extent different cognitive domains in pediatric patients with non-progressive ABI.

These conclusions should be considered with caution in view of the limitations of this study. First, the relatively small sample size implied limited power to detect any CCT effects, especially for secondary outcomes, where smaller effects were expected. Second, our stepped-wedge design did not include an active control group performing another training, thus hampering a controlled evaluation of the effects of specific training characteristics, such as therapist’s involvement or single- vs. multi-domain cognitive stimulation involvement. Third, due to unfortunate technical problems that occurred during execution of the study, the data from the computerized test used to assess attention and processing speed could not be collected for about half of the participants, thus hindering the analyses on these core cognitive domains and potentially masking other eventual benefits of the CCT. Fourth, only 5 out of the 50 brain-training games of the selected CCT available at the start of the study were chosen. This is because such games were considered to be easy to understand for children and feasible to be used also by patients with low intellectual functioning. However, such a choice may have limited the intensity of the cognitive stimulation. Thus, the conclusions of this work should only be referred to those games that the CCT used in this study and not to the format in which the CCT is provided in its commercial version. Finally, the short training duration could have limited the effects of the CCT on other cognitive abilities and the generalizability of CCT gains into more general life domains. Therefore, future studies are needed to better understand how intensive, how long and how specifically addressed to a single or to multiple cognitive domains a remote CCT should be to produce the best cognitive outcomes in children with non-progressive ABI.

## Methods

### Study design and procedure

The main study is registered with the ISRCTN registry, with study ID ISRCTN59250807 (https://www.isrctn.com/ISRCTN59250807?q=borgatti&filters=&sort=&offset=2&totalResults=2&page=1&pageSize=10&searchType=basic-search. Registration date: 25/10/2017). The trial is also registered with the Italian Ministry of Health Trial (44249 of 09/08/2016; approval: 17/11/2016). The study experimental protocol has been approved by the Ethical Committee of Scientific Institute, IRCCS E. Medea, Bosisio Parini, Italy (project number 284 on 01/03/2016, subsequently amended by project number 337 on 12/07/2016). Recruitment for this study started on 02/03/2016 and ended on 30/06/2017; follow-up assessments at T3 were concluded by 31/12/2017. Recruitment for the main study ended on 27/11/2019; the trial will end on 27/09/2020.

For this study, as for the main study, children were recruited from the brain damage registry of Scientific Institute, IRCCS E. Medea, Bosisio Parini, Italy. The referring clinician contacted by phone or email the parents of those children that fulfilled the inclusion criteria and proposed the research project giving advance notice of an ensuing contact by a member of the research team. Then, a researcher contacted parents by phone providing specific information on project objectives and methodology. Written informed consent was obtained by all parents agreeing to participate into the project, and all children provided their willingness to take part into the study. All procedures used for this study are in accordance with the 1964 Helsinki declaration and its later amendments and comparable ethical standards. All data were collected at Scientific Institute, IRCCS E. Medea, Bosisio Parini, Italy. This study was conducted in accordance with CONSORT guidelines for non-pharmacological interventions^76,77^.

A final sample of 60 patients was set for the main study in order to detect within-group change of moderate effect size (Cohen’s d = 0.47) with a power of 0.95 and the alfa level set at P < 0.05. The software G Power 3^78^ was used for this estimation. A post-hoc power analysis calculation was conducted for the present preliminary study on this subset of 32 participants, using the observed effect size to estimate the population effect size and to assess whether or not our statistical test had a fair chance of rejecting an incorrect null-hypothesis of no training effect.

The clinical trial applied a stepped-wedge research design, randomly assigning patients to two groups that differed for training and assessment timing^24^. Participant enrollment and randomization were conducted by a researcher of the Institute, who was not part of the research team responsible for testing participants. Randomization of patient assignment was based on a coin flip procedure using the randomization tool of Microsoft Excel: a random number was randomly associated to each recruited patient and determined the assignment to the Training-first Group (0.00 to 0.49) or the Waiting-first Group (0.50 to 1.00). The Training-first Group received the baseline assessment at T1 and then started the training; at T2, after training conclusion, it received the post-training evaluation; finally, at T3 it received the 2-month follow-up evaluation. The Waiting-first Group received the baseline assessment at T1, before starting a waiting-list period; at T2 it received the pre-training assessment and then started the training; finally, at T3, it concluded the training and received the post-training assessment. The research team was not blinded with respect to participants’ treatment allocation, while participants and outcome assessors were blinded.

In the main study, a 6-month follow-up assessment was scheduled for both the Training-first Group (T4) and the Waiting-first Group (T5). However, for this preliminary study we considered only the evaluations completed by the two groups at T3.

### Participants

To be eligible for the main study, participants had: i) to present a brain damage (congenital or acquired); ii) to be in chronic phase (at least 1 year after the event); iii) to be aged 11–16 years; iii) to speak Italian as a primary language. Exclusion criteria were: i) a previous diagnosis of psychiatric or cognitive problems (only for children with ABI); ii) severe visual, auditory or motor deficits that could interfere with training execution and outcome assessment; iii) undergoing a parallel cognitive rehabilitation treatment; ii) a diagnosis of photosensitive epilepsy, as a computer-based stimulation may produce negative health effects.

Specific inclusion criteria of this preliminary study were: i) to have suffered from a non-progressive ABI (e.g., TBI, stroke, anoxia, meningitis, encephalitis, post-surgical meningioma and acoustic neuroma)^79^, thus excluding patients with ABI due to brain tumor, which may present illness degeneration and/or of progressive neuroanatomical damage associated with adjuvant therapies; ii) to have concluded the research step T3 at 31^st^ December 2017.

Participants with TBI, anoxia or cerebral infection had a score < 9 on GCS (severe brain injury), while patients with stroke had a heterogeneous injury severity level (severe, moderate or mild brain injury). Brain injury severity (t(30) = −0.07, P = 0.94) and diagnosis (*χ*^2^(3) = 2.86, P = 0.41) were balanced between the two groups involved in this study, Group 1 (Training-first Group) and Group 2 (Waiting-firts Group) (Table 1).

Recruitment was deliberately not based on specific FSIQ thresholds or cognitive deficits in order to provide generalizable data for children with brain damage, who display different injury severity levels and cognitive functioning. On one hand, the choice to enroll also children with low intellectual ability was sustained by the fact that previous research on pediatric ABI highlighted the need to implement cognitive interventions also for patients with moderate and severe cognitive deficits^80^. Thus, in contrast with previous studies, which often included children with intellectual functioning out of the clinical range^25,81–85^, here we expanded the target population of children and adolescents with ABI. On the other hand, the decision to include also children having high average and superior FSIQ scores was based on data of previous research suggesting that CCT may boost neuropsychological performance in children with a neurodevelopmental disorder irrespective of the presence of general learning difficulties or cognitive impairments^86,87^. This was further corroborated by other previous studies indicating that a CCT may cause an increase in cortical thickness also in healthy individuals^88^, thus suggesting that such an intervention may likewise enhance the functional reorganization of neural networks in pediatric patients with non-progressive ABI with spared intellectual abilities.

### Intervention

The CCT used for this study was Lumosity Cognitive Training^TM^ ^39^, a web-based platform providing game-like exercises aimed at stimulating the following cognitive domains: memory, attention, cognitive flexibility, speed and problem-solving. Among the available Lumosity exercises, 5 games were chosen for this study (Table 3), each stimulating one of the target cognitive domains. Each game was proposed twice a day for a total of 10 daily exercises. As the CCT platform was in the English language and not in participants’ mother tongue, the selected games were also chosen based on the criterion that they relied on visual-spatial but not verbal information. Moreover, as this study involved children with heterogeneous cognitive functioning, we selected games that were easy to understand and perform, in order to allow children with low cognitive functioning to succeed in completing the training. At the same time, the capacity of the CCT to automatically adjust game complexity based on patients’ performance was thought to maintain motivation of patients with high intellectual functioning, as game demands became progressively more challenging. Moreover, the adaptation of training difficulty to patients’ abilities has been hypothesized to positively contribute to the activation of neuroplastic processes^89^. The training was performed at home. No feedback on cognitive performance was provided: the supervision of a clinician was only aimed at sustaining training adherence and motivation and recording the reasons of any eventual drop-outs. A weekly phone-based contact between the family and the clinician was scheduled with this aim.

**Table 3:** Description of the training games.

Game	Trained cognitive function(s)	Game rules and objectives
*Disillusion*	Cognitive flexibility	The patients are requested to insert a form in a matrix, matching it by symbol or color with another form, in light of the orientation of the target form (horizontal or vertical). This exercise trains the ability to respond to a task modifying the rule of matching on the basis of contextual information (cognitive flexibility). The more forms the patients are able to match, the higher is the score.
*Tidal Treasure*	Visual-spatial memory	The patients are presented with a beach where different objects appear. They have to select an object and then all objects are covered. In the subsequent screen, they are requested to select an object that is different from the previous one and so on. Each session is composed of three beaches. Patients fail when they select a stimulus that has been already chosen. The more objects the patients select, the higher is the score. This game trains visual-spatial memory.
*Speed Match*	Processing speed and visual-spatial memory	The patients have to indicate as quickly as possible whether a stimulus matches the last one displayed, based on the symbol presented on it. As speed performance improves, the number of trials increases, augmenting the level of difficulty. The more correct answers are given, the higher the score. This game trains processing speed and visual-spatial memory.
*Lost in Migration*	Selective attention	The patients are asked to indicate with the correct arrow key the direction of the central bird among a bird flock. Other birds are presented with the same or different direction from the central bird. The more correct answers are given, the higher the score. This game trains selective visual-spatial attention.
*Raindrops*	Arithmetic calculation	The patients are requested to solve mathematical operations contained in rain-drops. They are asked to give an answer before the raindrop falls into the sea at the bottom of the screen. They are presented with three game possibilities within each session. The more correct calculations are performed, the higher the score. This game trains arithmetic calculation.

Participants entered the program by inserting a personal email and password, which had been provided to them by the research team during the demonstration session. Children were required to perform the selected games twice a day (for a total of 10 daily exercises), for an average daily training duration of about 20 minutes. A total of 40 sessions were scheduled for each participant, to be performed 5 times a week in 8 weeks. The intensity and duration of this version of Lumosity Cognitive Training were chosen by our research team for this specific research project, based on extant literature on characteristics of CCT programs for childhood populations with brain damage or neurodevelopmental disorders.

### Measures

All instruments used to assess cognitive outcomes involved tasks with setting and stimuli different from the ones proposed by the CCT, in order to assess whether benefits from the training occurred in the general trained domains (near-transfer effects on tasks different from the training) and were not solely based on practice-related effects (engagement). All the outcome measures have been frequently used in previous research, constituting well-known tools of assessment. Moreover, outcome measures were all standardized, which granted more reliability to results.

#### Primary cognitive outcome

Visual-spatial working memory: the visual-spatial working memory span of the Corsi block tapping test^57^ was the primary outcome of the study. Indeed, all CCT games required the processing of visual-spatial information and 2 of them significantly relied on visual-spatial memory abilities. In the Corsi block tapping test children were asked to indicate a visual-spatial sequence on spatial separated blocks glued on a tablet, in the same order as it was presented by an examiner. Different block-tapping series of increasing length were presented and 3 trials per series were provided. The memory span corresponded to the maximum length of the series in which at least 2 trials were correctly indicated by children.

#### Secondary cognitive outcomes

- Cognitive flexibility: the computerized version of Wisconsin Card Sorting Test (WCST)^58^ was administered to test cognitive flexibility. Children were asked to identify a rule for associating cards and then to modify this rule in a flexible way on the basis of a computerized feedback. The number of total errors, consisting of the sum of perseverative and non-perseverative errors, was considered as measure of cognitive flexibility for this study.
- Problem-solving abilities: an age-appropriate problem-solving task and an arithmetic calculation task of the Italian battery AC-MT^59–61^ were used to test mathematical abilities. The problem-solving task required patients to solve 10 written problems involving reasoning and arithmetic abilities. The arithmetic calculation task required patients to solve 4 (for children of middle school) or 8 (for children of high school) math operations in a maximum time amount of 60 seconds each. A conventional score of 0 for the accuracy parameter and the maximum allowed time for solving operations were attributed to patients who were not able to perform the proposed arithmetic operations. Patients who were not able to be administered the age-appropriate arithmetic calculation task were thus assigned a conventional score of 0 on the age-appropriate problem-solving task. It is useful to clarify that, while the arithmetic calculation task represented a near-transfer measure, as it proposed a task similar to the Raindrops CCT game, the problem-solving task allowed assessing far-transfer effects, as it required more complex reasoning data and no similar task was provided by the CCT. Italian normative data were adopted to determine standardized patients’ scores on these tests.

The original research protocol of the main study included the assessment of attention and processing speed through the indexes omissions, commissions and HRT of the Conners’ Continuous Performance Test III (CPT-3)^90^. However, due to unfortunate technical issues occurred since March 2017, we could not record the results of half of the participants on CPT III at post-test and/or at 2-month follow-up and, thus, we excluded these measures from statistical analyses.

All cognitive measures collected were converted as z scores (Mean (M) = 0, standard deviation (SD) = 1), based on age-corrected normative data.

#### Secondary adjustment outcomes

- Psychological adjustment: the internalizing, externalizing and total scores of the Child Behavior Checklist 6–18 (CBCL)^62^ were considered for this far-transfer outcome. The CBCL is a 113-item questionnaire delivered to parents to assess psychological and adjustment problems of their children, by evaluating their responses on a 0–2 Likert scale. CBCL scores were expressed as T-scores (M = 50, SD = 10).

#### Covariates

We considered the following covariate measures:

- Improvement on CCT tasks (practice-related improvement): the Lumosity Performance Index (LPI), which was automatically supplied by the web-platform of the CCT, was used as a measure of improvement with respect to CCT tasks. This index assessed the average level of performance across training games. This measure was age-adjusted, but it was not standardized. The improvement on training tasks was calculated as the difference in LPI between the last and the first day of training (LPI-change).
- Full Scale Intelligence Quotient (FSIQ): global intelligence was assessed at baseline evaluation through Wechsler Intelligence Scales Fourth Edition (WISC-IV)^56^. FSIQ has a M of 100 and a SD of 15.

### Statistical analyses

Demographic, clinical and neuropsychological variables were described through descriptive statistics. T-test and χ^2^ were used to assess differences between the Training-first Group and the Waiting-first Group at baseline for continuous and categorical variables, respectively.

A modified intention to treat analysis approach was used, including in the analysis all the participants that had undergone the pre- and post-treatment evaluation sessions, even if they had not completed all the CCT sessions; no imputation of missing data was used, considering the limited sample size and observation points^91^. For each outcome measure, we calculated the change between T1 and T2 (delta 1) and between T2 and T3 (delta 2), measuring the difference between the second and the first time point. For the Training-first Group, delta 1 represents the treatment effect, while for the Waiting-first Group it represents the spontaneous change. In contrast, delta 2 represents the spontaneous change in the Training-first Group and the treatment effect in the Waiting-first Group. It was expected that delta 1 would be significantly higher in the Training-first Group than in the Waiting-first Group, and that delta 2 would be significantly higher in the Waiting-first Group than in the Training-first Group. This pattern of results would indicate that in either group the treatment effect was greater than spontaneous change.

Delta measures were entered into a series of 2 × 2 mixed ANOVAs to compare the change between T1 and T2 (delta 1) and between T2 and T3 (delta 2) in the two groups. Delta time (delta 1 or delta 2) was the within-subject variable and Group (Training-first or Waiting-first) was the between-subject factor. Furthermore, since the study involved patients with heterogeneous cognitive levels, results were controlled for the possible influence of individual intellectual ability and of practice-related improvements on the trained tasks. This allowed verifying whether the CCT could be more useful for patients with intellectual abilities falling into a specific range or whether the benefits on cognitive tests and psychological adjustments were solely associated with practice-related improvement in training game performance. Thus, whenever the main ANOVA showed significant interaction effects, we considered the FSIQ at baseline and the change of LPI between the first and the last training session (LPI-change) as covariates in follow-up ANCOVA analyses. No Bonferroni correction procedure was used for the ANOVA effects, due to the preliminary nature of the study. The Duncan correction procedure was used to control for multiple testing in post-hoc, pair-wise comparisons.

Furthermore, for those measures that resulted to be enhanced by the training, we run follow-up, pair-wise comparisons using dependent-sample t-tests (one tailed) to evaluate whether the treatment effects in the whole group were greater than spontaneous change between T2 and T3 in the Training-first Group and between T1 and T2 in the Waiting-first Group. Finally, in the Training-first Group the long-lasting effects of the training were assessed by comparing with dependent sample t-tests (one tailed) the 2-month-follow-up and the baseline scores (i.e., T3 vs T1) of the measures showing a significant improvement after the training (i.e., T2 vs T1). One-tailed tests were used for these follow-up analyses, as they were based on the results of the main ANOVAs showing performance improvement across time and we did not expect a worsening of the scores as compared to baseline.

Significance threshold was set at P < 0.05 for all tests. Effects sizes were reported as partial eta squared $({\eta }_{p}^{2})$ for the ANOVA effects^92^ and as Cohen’s d^93^ for the follow-up pairwise comparisons and interpreted according to standard benchmarks.

## Supplementary information

Research Protocol.

Consort Checklist.

## Data Availability

The dataset analyzed during the current study is available from the corresponding author on reasonable request.
